# Integrated Molecular Classification of Endometrial Cancer: Impact on Diagnosis, Prognosis, and Clinical Management—A Systematic Review

**DOI:** 10.3390/cancers18203316

**Published:** 2026-10-14

**Authors:** Silvio Antonio Galeano Reyes, María Eugenia Fraile Bethencourt, Serena Lacerenza, Carmen Camacho García, José Ernesto Moro Rodríguez, Pablo Bueno Ortiz, Víctor Macarrón Aguilera, Ivan Domazetovski, Miguel Andújar Sánchez

**Affiliations:** 1 Department of Pathological Anatomy, Complejo Hospitalario Universitario Insular-Materno Infantil de Gran Canaria, 35016 Las palmas de Gran Canaria, Spainslacere@gobiernodecanarias.org (S.L.); pbueort@gobiernodecanarias.org (P.B.O.); mandsan@gobiernodecanarias.org (M.A.S.); 2 Fundación Instituto de Investigación Sanitaria de Canarias (FIISC), 35012 Las palmas de Gran Canaria, Spain; 3 Instituto de Investigación Sanitaria de Canarias (IISC), 35019 Las Palmas de Gran Canaria, Spain; 4 Pathological Anatomy Area, Universidad Rey Juan Carlos, 28922 Madrid, Spain; joseernesto.moro@urjc.es

**Keywords:** endometrial cancer, molecular classification, TCGA, prognosis, FIGO 2023 staging, personalized medicine

## Abstract

Objective: To systematically review the literature on the impact of integrated molecular classification of endometrial cancer on diagnosis, prognostic stratification, and clinical management. Methods: A systematic search was conducted in PubMed/MEDLINE, Web of Science, Scopus, and Cochrane Library (January 2013–December 2025), following PRISMA 2020 guidelines. We included original studies, systematic reviews, clinical guidelines, and cost-effectiveness analyses applying TCGA molecular classification or its surrogate classifiers (ProMisE, TransPORTEC). Data were extracted on subtype distribution, survival outcomes, hazard ratios, stage migration, and therapeutic implications. Results: Fifty studies were included. Of these, 13 provided data on molecular subtype distribution (comprising 3527 patients), 8 reported survival outcomes, and 7 reported hazard ratios. The weighted subtype distribution from the 13 studies was as follows: POLEmut 8.9%, MMRd 25.8%, p53abn 16.7%, and NSMP 48.2%. POLEmut was associated with an excellent prognosis (5-year overall survival >95%, hazard ratio <0.5), while p53abn represented the poorest prognosis group (5-year disease-free survival 33–58%, hazard ratio >2.0). p53abn was the only independent risk factor for peritoneal (odds ratio 8.54) and vaginal (odds ratio 6.51) recurrence. Molecular classification modified risk group assignment in 6–33% of patients and improved prognostic discrimination of the 2023 FIGO staging system. Therapeutic implications include treatment de-escalation in POLEmut, immunotherapy in MMRd, and intensive chemotherapy in p53abn. Barriers to implementation include access to POLE sequencing and cost-effectiveness. Conclusions: Integrated molecular classification is an essential tool for prognostic stratification and treatment personalization in endometrial cancer. Molecular subtypes demonstrate clearly differentiated prognostic profiles and recurrence patterns, justifying their systematic incorporation into clinical practice and international guidelines.

## Simple Summary

Endometrial cancer is the most common cancer of the uterus, and its incidence is rising worldwide. Traditionally, doctors have decided how to treat this cancer based on how the tumor looks under a microscope and how far it has spread. However, tumors that appear similar can behave very differently. Scientists discovered that endometrial cancer can be divided into four distinct groups based on the genetic changes inside the tumor. This review summarizes evidence from 50 studies, including more than 3500 patients, to understand how this new classification system affects diagnosis, prognosis, and treatment. We found that one group (called POLEmut) has an excellent prognosis and may not need aggressive treatment, while another group (p53abn) has the worst prognosis and requires intensive therapy. The other two groups (MMRd and NSMP) have intermediate outcomes, with MMRd patients benefiting from immunotherapy. This classification also changes the risk category for up to one-third of patients. Our findings support the routine use of molecular classification in all patients with endometrial cancer to personalize treatment and improve outcomes.

## 1. Introduction

### 1.1. Key Messages

What is already known on this topic:

The 2013 TCGA study identified four molecular subtypes of endometrial cancer (POLEmut, MMRd, p53abn, NSMP) with distinct prognostic profiles, but implementation in routine clinical practice has been limited by technical complexity and cost.

What this study adds:

This systematic review synthesizes evidence from 50 studies, with 13 providing detailed molecular subtype data from 3527 patients, confirming the independent prognostic value of molecular classification, demonstrating subtype-specific recurrence patterns (peritoneal/vaginal for p53abn; distant for NSMP), and quantifying the impact on risk reclassification (6–33% of patients) with the new FIGO 2023 staging.

How this study might affect research, practice or policy:

Our findings support systematic implementation of molecular classification in all endometrial cancer patients, guide personalized adjuvant therapy (de-escalation for POLEmut, immunotherapy for MMRd, intensive chemotherapy for p53abn), and highlight the need for NSMP subclassification and validation of FIGO 2023 in prospective cohorts.

### 1.2. Introduction

Endometrial cancer has an increasing global incidence due to population aging and rising obesity rates [1,2,3]. In 2020, over 417,000 new cases and approximately 97,000 deaths were reported worldwide [4]. Although most patients (70–80%) are diagnosed at early stages (FIGO I-II) with 5-year overall survival exceeding 80%, a high-risk subgroup accounts for the majority of mortality, with survival rates dropping below 50% in advanced stages [5,6].

For decades, prognostic stratification of endometrial cancer relied on conventional clinicopathological parameters. In 1983, Bokhman proposed a dichotomous classification distinguishing type I (endometrioid, estrogen-dependent, favorable prognosis) from type II (non-endometrioid, estrogen-independent, poor prognosis) tumors [7]. Although conceptually useful, this model demonstrated limited reproducibility and inability to capture biological complexity, particularly in tumors with ambiguous or mixed morphology [8]. Similarly, the International Federation of Gynecology and Obstetrics (FIGO) staging systems of 1988 and 2009 incorporated histologic type, tumor grade, myometrial invasion depth, and nodal involvement, but remained fundamentally anchored to anatomic criteria [9]. These tools, while clinically useful, have well-documented limitations, including poor interobserver reproducibility in grade and histotype assignment, especially in high-grade tumors [10,11].

The classification paradigm for endometrial cancer underwent a radical transformation in 2013 with publication of The Cancer Genome Atlas (TCGA) project results [12]. Through integrated analysis of 373 endometrial carcinomas including whole-exome sequencing, copy number analysis, methylation profiles, and gene expression, TCGA identified four intrinsic molecular subtypes with clearly differentiated genomic and prognostic profiles:

1. POLE ultramutated (7–10%): characterized by pathogenic mutations in the exonuclease domain of polymerase ε, extremely high mutation burden, and excellent prognosis independent of histologic grade [12,13].
2. Microsatellite instability (MSI)/mismatch repair deficiency (MMRd) (25–30%): hypermutated phenotype with intermediate prognosis [12].
3. Copy-number low (CNL) (40–50%): subsequently termed “no specific molecular profile” (NSMP), grouping tumors without defining molecular alterations with intermediate prognosis [12].
4. Copy-number high (CNH) (15–20%): characterized by TP53 mutations (p53abn), genomic instability, and the worst prognosis of all subtypes [12].

This classification demonstrated for the first time that the biological behavior of endometrial cancer is determined by molecular alterations that transcend morphology, explaining why some high-grade tumors (such as certain POLEmut) have indolent evolution, while others of low grade (such as some p53abn) may behave aggressively [13,14].

Direct application of TCGA classification in clinical practice was limited by technical complexity and high cost. In response, simplified but highly concordant approaches emerged, such as the ProMisE (Proactive Molecular Risk Classifier for Endometrial Cancer) classifier and the TransPORTEC consortium proposals [15,16,17,18]. These strategies combine immunohistochemistry (for MMR and p53 protein evaluation) with targeted sequencing of the POLE exonuclease domain, enabling reliable assignment of the four molecular subtypes with high concordance with the original TCGA (>90%) [15,16,17,18]. Validation of these classifiers in multiple independent cohorts and their ability to stratify risk more precisely than classic clinicopathological factors led to their incorporation into major clinical practice guidelines. Thus, the 2021 joint European Society of Gynecological Oncology (ESGO), European Society for Radiotherapy and Oncology (ESTRO), and European Society of Pathology (ESP) guideline integrated molecular classification into prognostic risk group definitions and adjuvant treatment recommendations [6]. Subsequently, the 2023 FIGO staging update went further by incorporating (optionally) molecular subtypes as stage modifiers in early disease (stages IAm-POLEmut and IICm-p53abn), explicitly recognizing that tumor biology may override anatomic extent in certain contexts [19].

Despite these advances, widespread implementation of integrated molecular classification raises numerous questions and challenges. Uncertainties persist regarding applicability across all histologic subtypes (especially non-endometrioid carcinomas), real impact on therapeutic decision making, management of cases with multiple molecular classifiers, cost-effectiveness in different healthcare settings, and optimal integration with classic clinicopathological parameters [20,21]. In this context, an updated and critical synthesis of available evidence is essential to evaluate the true impact of this new classification on diagnosis, prognosis, and clinical management of patients with endometrial cancer.

Despite the growing body of research on molecular classification of endometrial cancer, most previous reviews have focused on specific aspects, such as prognostic value or distribution of molecular subtypes. However, to our knowledge, no previous systematic review has comprehensively integrated data on molecular subtype distribution, prognostic stratification, subtype-specific recurrence patterns, stage migration under the new FIGO 2023 system, therapeutic implications, and barriers to clinical implementation in a single analysis. Furthermore, the recent publication of multiple studies evaluating the clinical impact of molecular classification—particularly regarding recurrence patterns and risk reclassification—warrants an updated and critical synthesis. The present systematic review aims to fill this gap by providing a comprehensive and up-to-date overview of the current state of integrated molecular classification of endometrial cancer and its implications in daily clinical practice.

The present systematic review aims to analyze and synthesize the literature published over the last decade to provide a comprehensive overview of the current state of integrated molecular classification of endometrial cancer and its implications in daily clinical practice.

## 2. Methods

### 2.1. Study Design

We conducted a systematic literature review following the Preferred Reporting Items for Systematic Reviews and Meta-Analyses (PRISMA) 2020 guidelines [22]. The review protocol was not registered in PROSPERO. The completed PRISMA 2020 checklist is provided in Table S3 (Supplementary Materials).

### 2.2. Search Strategy

We performed a systematic search in the following electronic databases: PubMed/MEDLINE, Web of Science (Core Collection), Scopus, and Cochrane Library. The search was conducted in December 2025 and covered the period from January 2013 (year of TCGA publication) to December 2025.

The search strategy combined Medical Subject Headings (MeSH) terms and free-text terms using Boolean operators AND/OR. The detailed PubMed strategy (adapted for other databases) was as follows:

(“endometrial neoplasms”[MeSH] OR “endometrial cancer”[tiab] OR “endometrial carcinoma”[tiab] OR “endometrial tumor”[tiab]) AND (“molecular classification”[tiab] OR “molecular subtyping”[tiab] OR “TCGA”[tiab] OR “The Cancer Genome Atlas”[tiab] OR “ProMisE”[tiab] OR “POLE”[tiab] OR “p53”[tiab] OR “TP53”[tiab] OR “MMRd”[tiab] OR “mismatch repair”[tiab] OR “MSI”[tiab] OR “microsatellite instability”[tiab] OR “NSMP”[tiab] OR “copy number”[tiab]) AND (“prognosis”[MeSH] OR “survival”[tiab] OR “recurrence”[tiab] OR “risk stratification”[tiab] OR “FIGO 2023”[tiab] OR “ESGO”[tiab] OR “adjuvant treatment”[tiab] OR “immunotherapy”[tiab]).

Additionally, we reviewed reference lists of included articles and relevant narrative reviews to identify additional studies not captured by the electronic search.

### 2.3. Eligibility Criteria

Inclusion criteria: We included original studies (prospective or retrospective cohorts), validation studies, clinical trials (including ancillary analyses), systematic reviews with or without meta-analyses, clinical practice guidelines, and consensus documents. Studies were required to have patients with histologically confirmed endometrial cancer of any histologic type and FIGO stage, applying TCGA molecular classification or surrogate classifiers (ProMisE, TransPORTEC), including determination of at least three of four subtypes (POLEmut, MMRd, p53abn, NSMP). Studies reporting data on molecular subtype distribution, survival (overall survival, disease-free survival, recurrence-free survival, disease-specific survival), hazard ratios, risk stratification changes, stage migration (especially with FIGO 2023), therapeutic implications, cost-effectiveness analyses, or implementation barriers were included. Articles were limited to English or Spanish language, published from January 2013 to December 2025.

Exclusion criteria: Editorials, letters to the editor, comments without original data, case reports or series with fewer than 10 patients, studies in animal models or cell lines (unless providing relevant translational data), studies not clearly differentiating molecular subtypes or analyzing only one subtype in isolation, and articles not available in full text were excluded.

### 2.4. Study Selection Process

Search results were exported to a bibliographic manager for duplicate removal. The selection process was conducted in two phases:

Phase 1 (title/abstract screening): Two reviewers independently examined titles and abstracts of all identified articles. Clearly irrelevant articles were excluded. Disagreements were resolved by consensus or through the involvement of a third reviewer.

Phase 2 (full-text review): Full texts of potentially eligible articles were retrieved. Two reviewers independently assessed eligibility according to predefined criteria. Reasons for the exclusion of studies not passing this phase were documented.

The selection process is represented by the PRISMA 2020 flow diagram (Figure 1).

### 2.5. Data Extraction

A standardized data-extraction sheet was designed in Microsoft Excel. For each included study, two reviewers independently extracted the following information:

- Study characteristics: author, year of publication, country (or countries), study design, recruitment period, number of centers, and total sample size.
- Population characteristics: age, body mass index, histologic type, FIGO grade, FIGO stage (2009 and/or 2023), presence of lymphovascular invasion, and nodal status.
- Molecular classification: determination method (immunohistochemistry, next-generation sequencing, targeted sequencing), number and percentage of patients in each subtype (POLEmut, MMRd, p53abn, NSMP), and frequency of multiple classifiers.
- Survival outcomes: 5-year survival rates (overall survival, disease-free survival, recurrence-free survival, disease-specific survival) by molecular subtype, hazard ratios with 95% confidence intervals and *p*-values, and Kaplan–Meier curves.
- Stage migration and risk reclassification: percentage of global migration, upstaging, downstaging, and changes in ESGO/ESTRO/ESP risk group assignment.
- Therapeutic implications: treatment recommendations based on molecular profile, response to immunotherapy, chemotherapy, radiotherapy, and targeted therapies.
- Implementation barriers: data on cost-effectiveness, technology access, and interpretation limitations.

Discrepancies in data extraction were resolved through discussion and consensus.

### 2.6. Quality Assessment and Risk of Bias

Methodological quality of included studies was assessed using tools adapted to each study design:

- Cohort studies: Newcastle-Ottawa Scale [23], with scores ≥ 7/9 considered high quality.
- Randomized clinical trials (and ancillary analyses): Cochrane Collaboration Risk of Bias tool (RoB 2) [24].
- Systematic reviews and meta-analyses: AMSTAR-2 tool [25].
- Clinical practice guidelines: AGREE II instrument [26].
- Cost-effectiveness studies: CHEERS criteria [27].

Assessment was performed by two reviewers independently, with disagreements resolved by consensus. A comprehensive quality assessment and risk of bias table for all 50 included studies is provided in Table S1 (Supplementary Materials).

### 2.7. Data Synthesis

Given expected heterogeneity in study designs, populations, molecular classification methods, and reported outcomes, we performed narrative synthesis of findings. Results were structured according to specific review objectives, grouping studies by theme (subtype distribution, prognostic value, stage migration, therapeutic implications, etc.).

Summary tables were prepared presenting characteristics of included studies, molecular subtype distribution, and main survival outcomes. When possible, and data-permitting (sufficient homogeneity and hazard ratio availability), random-effects meta-analyses were performed, with heterogeneity assessed using the I^2^ statistic [28].

In accordance with the journal’s guidelines, we will provide our data for independent analysis by a selected team by the Editorial Team for the purposes of additional data analysis or for the reproducibility of this study in other centers if such is requested.

### 2.8. Ethics Statement

This systematic review was exempt from Institutional Review Board (IRB) approval as it involved no primary data collection from human subjects. All included studies had previously obtained appropriate ethical approval and informed consent as reported in their respective publications.

## 3. Results

A total of 50 studies met the eligibility criteria and were included in this systematic review. The PRISMA flow diagram (Figure 1) details the selection process. The methodological quality of all included studies is summarized in Table S1 (Supplementary Materials).

The majority of studies were published between 2024 and 2026 (81.4%), reflecting growing interest in endometrial cancer molecular classification in recent years. Retrospective cohort studies predominated (63.0%), followed by narrative reviews and position papers (20.4%). Geographically, Europe concentrated the largest number of publications (44.4%), with significant contributions from Italy [10,29,30], Spain [26], and the Netherlands [12], followed by Asia (24.1%) with relevant series from Japan [14,17,31] and South Korea [10,20], and North America (14.8%) [11,32,33,34]. Sample sizes of cohort studies ranged from 20 to over 130,000 patients, with the most frequent range being 100–500 patients (46.3%). Regarding molecular classification methodology, 70.4% of studies employed the ProMisE algorithm or similar approaches combining immunohistochemistry for MMR and p53 with targeted POLE sequencing [15,16,17,18], while 22.2% utilized massive sequencing following the original TCGA approach [12]. Finally, 27.8% of studies specifically evaluated the impact of the new FIGO 2023 staging system [19,20,34,35].

Of the 50 included studies, 13 provided data on the frequency of the four molecular subtypes, comprising a total of 3527 endometrial cancer patients (Table 1). The most frequent subtype was NSMP, with a weighted percentage of 48.2% (range: 26.1–67.0%), followed by MMRd at 25.8% (range: 18.4–39.1%), p53abn at 16.7% (range: 9.6–23.3%), and POLEmut at 8.9% (range: 0–21.7%). When the cohort that did not perform POLE sequencing (Lee et al., 2025 [36]) is excluded, the weighted frequency of POLEmut increases to 9.9% and the total number of patients included in the weighted analysis is 3182. These figures are consistent with those reported in the original TCGA series and in the large ProMisE validation studies [12,15,16,17,18].

Notable variability was observed in POLEmut frequency, ranging from 0% in a study that did not perform POLE sequencing [36] to 21.7% in a selected cohort of high-grade carcinomas [37]. The inclusion of cohorts without POLE sequencing may underestimate the true global incidence of POLEmut; when the Lee et al. (2025) [36] cohort, which explicitly did not perform POLE sequencing, is excluded, the weighted frequency of POLEmut increases from 8.9% to 9.9%. This variability reflects both differences in study populations and methodologies employed for molecular classification.

Eight studies evaluated patient survival stratified by molecular subtype (Table 2). The results consistently confirm the excellent prognosis of the POLEmut subtype, with 5-year overall survival rates ranging from 95% to 100%, even in the absence of adjuvant therapy in early stages [14,19,38]. In contrast, the p53abn subtype is associated with the worst prognosis, with 5-year disease-free survival rates dropping to 33–43% in some series [10,38,39]. MMRd and NSMP subtypes show intermediate behavior, with 5-year overall survival around 80–90% and 78–90%, respectively [38,39].

Seven studies (including one meta-analysis with over 4700 patients) reported hazard ratios quantifying risk associated with each molecular subtype (Table 3). Results consistently identify the p53abn subtype as having the worst prognosis, with hazard ratios ranging from 1.76 to 8.54 for different outcomes, including peritoneal, vaginal, distant recurrence, and worse overall survival [38,39,40]. In the meta-analysis by Yin and colleagues [40], the copy-number high (p53abn) subtype showed strong association with advanced stage (odds ratio 3.05), presence of lymphovascular invasion (odds ratio 2.12), and nodal metastases (odds ratio 3.06).

Conversely, POLEmut was confirmed as a protective factor, with hazard ratios below 0.5 in most analyses, including lower probability of advanced stage (odds ratio 0.39) and absence of nodal metastases (odds ratio 0.25) [40]. MMRd and NSMP subtypes showed intermediate behavior, though with nuances: while MMRd showed no significant association with advanced stage in the meta-analysis (hazard ratio 1.04; 95% confidence interval 0.88–1.23) [40], NSMP was associated with higher risk of distant recurrence in the Luzarraga study (hazard ratio 2.35; 95% confidence interval 1.1–5.0) [38].

A forest plot-style figure (Figure 2) displays the individual study estimates and pooled estimates from the meta-analysis by Yin et al. (2025) [40], providing a visual representation of the effect sizes and their confidence intervals.

A novel finding from our review was the identification of subtype-specific recurrence patterns. The study by Luzarraga and colleagues [38] demonstrated that p53abn was the only independent risk factor for peritoneal (odds ratio 8.54; 95% confidence interval 2.0–36.3) and vaginal (odds ratio 6.51; 95% confidence interval 1.1–37.4) recurrence, while NSMP was associated with distant recurrence (hazard ratio 2.35; 95% confidence interval 1.1–5.0). These findings have direct implications for personalized surveillance protocols.

Incorporation of molecular classification modifies risk group assignment in 6–33% of patients, predominantly toward lower-risk groups due to the identification of POLEmut. The new FIGO 2023 staging system, which incorporates molecular modifiers (IAm-POLEmut and IICm-p53abn), demonstrates improved prognostic discrimination compared to the previous 2009 system [19,20,35,41]. External validation studies have confirmed these findings in independent cohorts [42]. A summary of the key findings of this systematic review is presented in Table 4.

## 4. Discussion

### 4.1. Summary of Main Results

This systematic review, including 50 studies (with 13 providing molecular subtype data from 3527 patients, 8 reporting survival outcomes, and 7 reporting hazard ratios), provides comprehensive evidence confirming the independent prognostic value of integrated molecular classification and its impact on clinical management. The primary findings demonstrate that NSMP is the most frequent subtype (48.2%), followed by MMRd (25.8%), p53abn (16.7%), and POLEmut (8.9%), consistent with original TCGA series and large ProMisE validation studies [12,15,16,17,18]. The prognostic value of each subtype is clearly differentiated: POLEmut shows excellent prognosis (5-year overall survival >95%, hazard ratio <0.5), while p53abn represents the poorest prognosis group (5-year disease-free survival 33–58%, hazard ratio >2.0 for multiple outcomes), with MMRd and NSMP demonstrating intermediate and more heterogeneous behavior [10,14,38,39].

Regarding recurrence patterns, p53abn is the only independent risk factor for peritoneal (odds ratio 8.54) and vaginal (odds ratio 6.51) recurrence, while NSMP and p53abn predict distant recurrence [38]. Molecular classification modifies risk group assignment in 6–33% of patients and improves prognostic discrimination with the new FIGO 2023 staging system [19,20,34,35,41]. The therapeutic implications are substantial, enabling personalized adjuvant treatment: de-escalation in POLEmut, immunotherapy in MMRd, intensive chemotherapy in p53abn, and potentially targeted therapies in NSMP subgroups (PIK3CA, estrogen receptor, L1CAM) [32,33,43,44].

### 4.2. Results in the Context of the Published Literature

Our results are consistent with major reference studies. In the TCGA analysis, Kandoth and colleagues [12] first described the four molecular subtypes and their association with survival, findings subsequently validated in independent cohorts through the ProMisE classifier [15,16,17,18] and in the PORTEC-3 clinical trial [29]. Regarding subtype distribution, Raffone and colleagues [31], in a meta-analysis including over 5000 patients, reported frequencies very similar to our findings: POLEmut 8%, MMRd 28%, p53abn 17%, and NSMP 47%. This concordance reinforces the robustness of our estimates.

Regarding prognostic value, the study by León-Castillo and colleagues [29] on the PORTEC-3 cohort demonstrated that patients with p53abn tumors derive the greatest benefit from adding chemotherapy to radiotherapy, while those with POLEmut have excellent prognosis even without adjuvant therapy. Our data, showing hazard ratio of 3.05 for advanced stage in p53abn and 0.39 for POLEmut [40], support these conclusions.

The identification of subtype-specific recurrence patterns represents a novel contribution. The study by Luzarraga and colleagues [38] is, to our knowledge, the first to demonstrate that p53abn is the only independent risk factor for peritoneal and vaginal recurrence, while NSMP is associated with distant recurrence. These findings have direct implications for personalized surveillance protocols.

It is important to emphasize that, although the prognostic value of molecular subtypes—particularly the excellent prognosis of POLEmut—has been consistently demonstrated since the original TCGA publication and subsequent ProMisE validation studies, the clinical utility of molecular classification extends beyond this well-established association. Our review provides novel contributions by integrating aspects that have not been jointly addressed in previous publications, including subtype-specific recurrence patterns, the quantification of risk reclassification under FIGO 2023, the management of multiple classifiers, and real-world implementation barriers. These elements are essential for translating molecular classification from a prognostic tool into a practical guide for personalized clinical management.

### 4.3. Strengths and Weaknesses

#### 4.3.1. Strengths

To our knowledge, this is the first systematic review to comprehensively integrate data on molecular subtype distribution, prognostic value, subtype-specific recurrence patterns, stage migration under the new FIGO 2023 system, therapeutic implications, and barriers to clinical implementation in a single analysis. While the association between POLEmut and excellent prognosis is well established, our review provides novel insights by (1) demonstrating subtype-specific recurrence patterns (peritoneal/vaginal for p53abn; distant for NSMP) with direct implications for personalized surveillance; (2) quantifying the impact of molecular classification on risk reclassification (6–33% of patients); (3) integrating real-world data on implementation barriers and cost-effectiveness; and (4) providing the most updated synthesis currently available, with 81.4% of included studies published between 2024 and 2026.

The inclusion of 50 studies (with 13 providing detailed molecular subtype data from 3527 patients) provides robustness to our estimates. We performed a detailed analysis of molecular subtypes, including hazard ratios and survival rates, and incorporated the most recent studies evaluating the impact of FIGO 2023 staging. Our methodology followed rigorous PRISMA 2020 guidelines with systematic quality assessment using validated tools.

The certainty of evidence for the main outcomes was assessed using the GRADE framework (Table S2, Supplementary Materials). The prognostic associations of POLEmut and p53abn were rated as having moderate certainty, while other outcomes (recurrence patterns, FIGO 2023 comparison, and MMRd/NSMP intermediate prognosis) were rated as having low certainty, primarily due to the observational nature of the included studies and the predominance of single-study evidence for some outcomes.

#### 4.3.2. Weaknesses

We acknowledge several limitations. There is heterogeneity across studies in designs, populations, molecular classification methods, and adjustment variables, which may affect comparability. Publication bias is possible despite comprehensive multi-database searching. Absence of individual patient data prevented more precise meta-analyses for all outcomes. Most studies originated from referral centers, potentially limiting generalizability to resource-limited settings. The restriction to English and Spanish language may have excluded relevant studies in other languages. Finally, the variable quality of included studies and different reporting standards for outcomes may have influenced our findings.

The weighted frequency of POLEmut (8.9% in the overall analysis) may be underestimated due to the inclusion of cohorts that did not perform POLE sequencing. When the Lee et al. (2025) [36] cohort, which explicitly did not perform POLE sequencing, is excluded, the weighted frequency increases to 9.9%. This highlights the importance of universal POLE sequencing for accurate molecular classification and underscores the need for standardized testing protocols across centers. Additionally, molecular classification has shown limited prognostic value in stage IV disease, as reported by Uijterwaal et al. [45], suggesting that its clinical utility may be restricted to early-stage disease. 

### 4.4. Implications for Practice and Future Research

The clinical implications of our findings are substantial. First, given its ability to stratify risk and guide treatment, molecular classification should be performed systematically in all endometrial cancer patients, as recommended by ESGO/ESTRO/ESP guidelines [6] and FIGO 2023 [19]. However, access barriers remain, particularly for POLE sequencing, and cost-effectiveness strategies are needed [34]. Real-world data from the United States show that molecular testing rates have increased over time but remain influenced by practice setting, insurance status, and disease stage [46]. Second, personalized adjuvant therapy can be guided by molecular subtype: POLEmut patients may be considered for treatment de-escalation even in early-stage high-grade disease [38]; MMRd tumors are candidates for immunotherapy both in first-line and recurrent disease [32,33]; p53abn requires intensive adjuvant chemotherapy independent of other clinicopathological factors [29,39]; and NSMP high-risk subgroups (estrogen receptor-negative, L1CAM-positive, CTNNB1 or PIK3CA mutations) will benefit from refined therapeutic indications in the future [43,44]. Prospective real-world implementation studies have demonstrated the feasibility of integrating next-generation sequencing and immunohistochemistry into routine clinical practice [47].

Third, different recurrence patterns by molecular subtype could translate into risk-adapted surveillance protocols, with increased vigilance for peritoneal and vaginal recurrence in p53abn and distant recurrence in NSMP [38]. In stage IV disease, cytoreductive surgery has been associated with improved survival, particularly in patients with p53abn and NSMP-estrogen receptor-negative tumors [48].

Several unanswered questions should be addressed in future research. Subclassification of the NSMP group through identification of additional biomarkers (estrogen receptor, progesterone receptor, L1CAM, CTNNB1, PIK3CA) will enable better prognostic stratification and identification of therapeutic targets in this heterogeneous group [43,44]. Recent evidence suggests that estrogen receptor status may refine prognostic stratification within high-grade NSMP tumors, although its independent prognostic value remains debated [49]. Management of multiple classifiers, although current guidelines recommend classifying these cases according to the best prognosis subtype [30], requires further confirmation. The role of immunotherapy in POLEmut tumors, theoretically beneficial due to high mutation burden, currently has limited evidence and requires specific clinical trials. Prospective validation of FIGO 2023 staging shows promising results [19,20,35] but requires longer follow-up series to confirm prognostic superiority. Finally, cost-effectiveness of universal implementation, especially in resource-limited settings, requires further study; selective testing strategies may be more efficient.

## 5. Conclusions

Integrated molecular classification of endometrial cancer, based on POLEmut, MMRd, p53abn, and NSMP subtypes, is currently an indispensable tool for prognostic stratification and therapeutic decision making, overcoming limitations of systems based exclusively on clinicopathological parameters.

Molecular subtype distribution from over 3500 patients confirms NSMP as the most frequent (48.2%), followed by MMRd (25.8%), p53abn (16.7%), and POLEmut (8.9%). Prognostic value is clearly differentiated: POLEmut is associated with excellent prognosis (5-year overall survival >95%, hazard ratio <0.5), justifying treatment de-escalation strategies, while p53abn represents the worst prognosis group (5-year disease-free survival 33–58%, hazard ratio >2.0), requiring intensive adjuvant therapy.

Recurrence patterns differ by molecular subtype, with p53abn as the only independent risk factor for peritoneal and vaginal recurrence, while NSMP and p53abn predict distant recurrence. Molecular classification modifies risk group assignment in 6–33% of patients and improves prognostic discrimination of the new FIGO 2023 staging system, confirming its superiority over the previous 2009 system.

Therapeutically, molecular classification enables treatment de-escalation in POLEmut, immunotherapy in MMRd, intensive chemotherapy in p53abn, and exploration of targeted therapies in NSMP subgroups. Barriers to universal implementation persist, particularly regarding POLE sequencing access, cost-effectiveness, and interpretation of multiple classifiers; however, selective molecular testing strategies may optimize resources without compromising stratification accuracy.

Current evidence supports the recommendation of major clinical guidelines (ESGO/ESTRO/ESP [6], ESMO [50], NCCN, FIGO 2023 [19]) to perform molecular classification systematically in all endometrial cancer patients. Further research is needed on NSMP subclassification, optimal management of multiple classifiers, and prospective validation of FIGO 2023 staging in independent cohorts with prolonged follow-up.

## Figures and Tables

**Figure 1:**
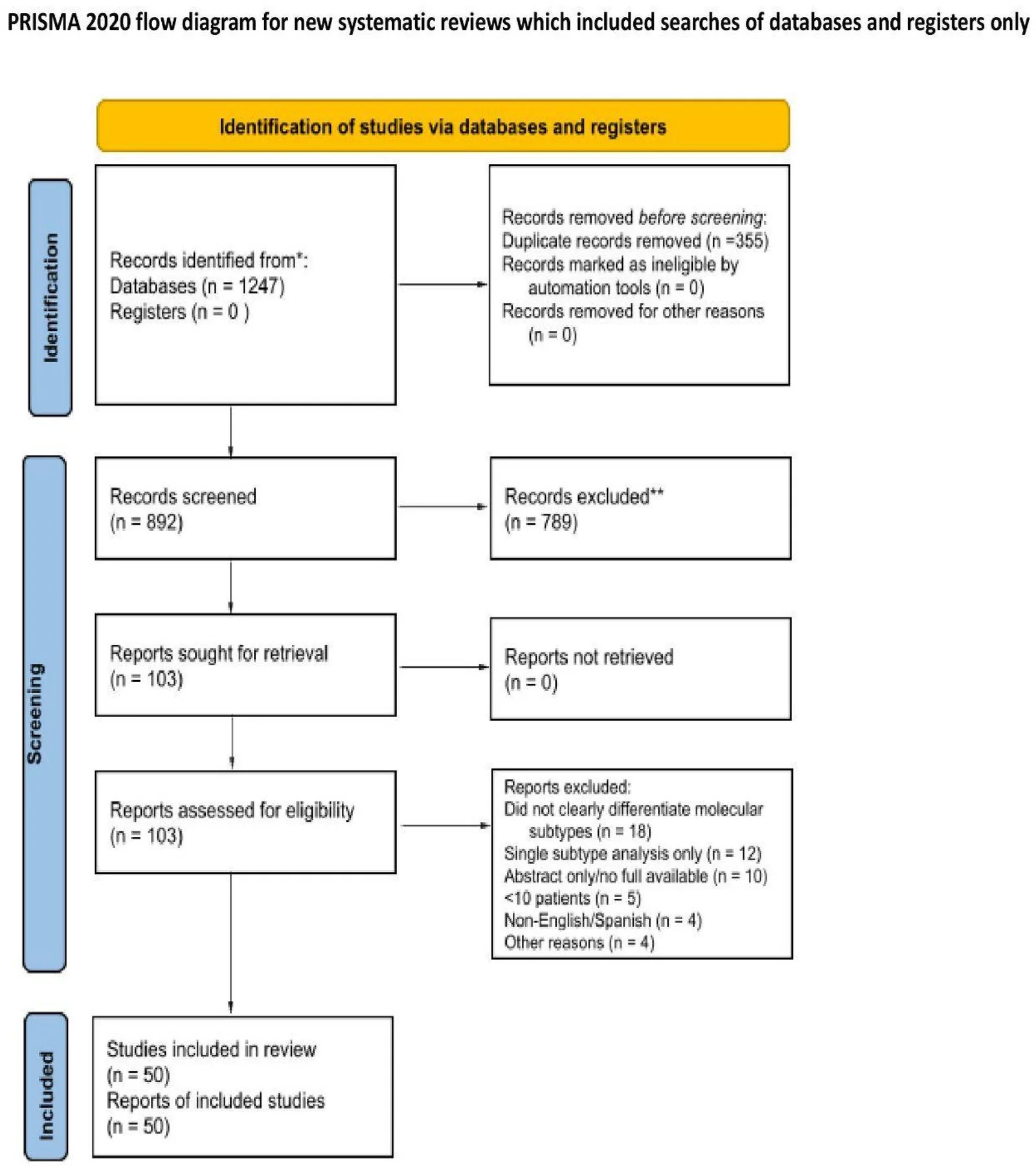
PRISMA 2020 Flow Diagram. * Consider, if feasible to do so, reporting the number of records identified from each database or register searched (rather than the total number across all databases/registers). ** If automation tools were used, indicate how many records were excluded by a human and how many were excluded by automation tools. Source: [22]. This work is licensed under CC BY 4.0. To view a copy of this license, visit https://creativecommons.org/licenses/by/4.0/ (accessed on 10 October 2026).

**Figure 2:**
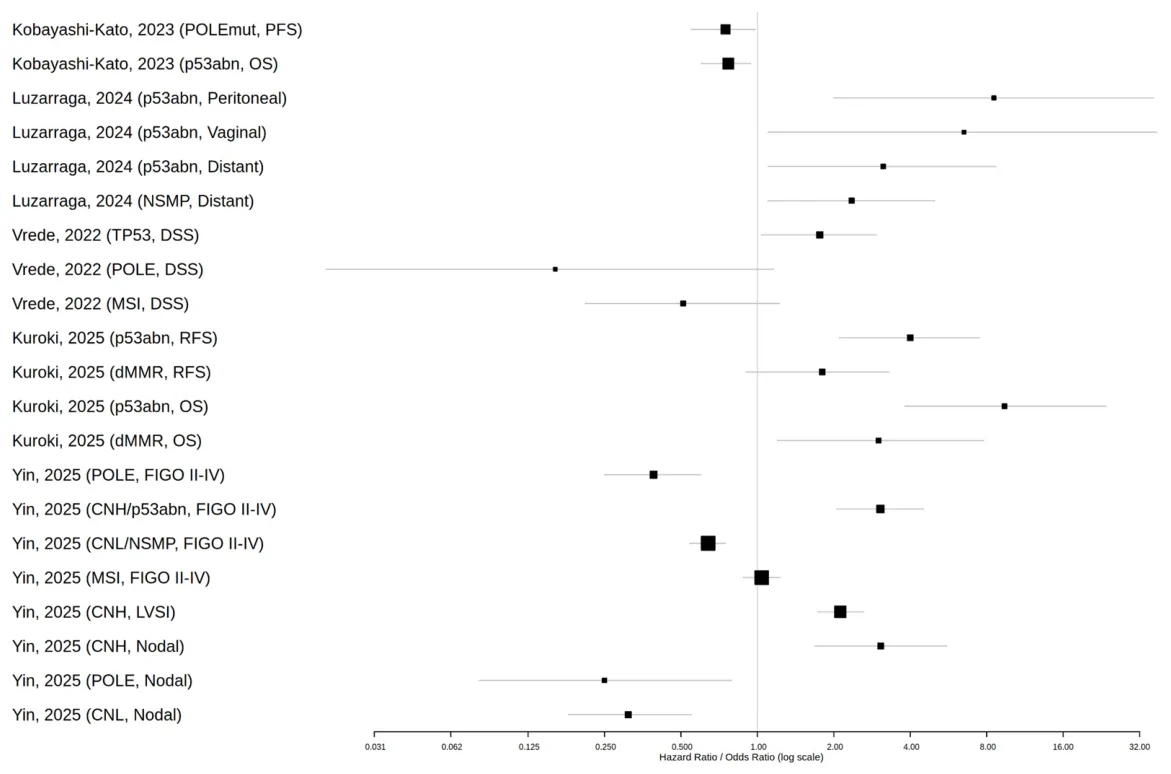
Forest plot of hazard ratios (HRs) and odds ratios (ORs) for recurrence and survival outcomes by molecular subtype. The plot displays individual study estimates from Kobayashi-Kato et al. [14], Luzarraga et al. [38], Vrede et al. [28], and Kuroki et al. [39], as well as pooled estimates from the meta-analysis by Yin et al. [40]. A vertical line at x = 1 indicates no effect. Values to the left of the line indicate a favorable prognosis (HR/OR < 1), while values to the right indicate a worse prognosis (HR/OR > 1). Horizontal lines represent 95% confidence intervals. Abbreviations: CI, confidence interval; CNH, copy-number high; CNL, copy-number low; dMMR, mismatch repair deficient; DSS, disease-specific survival; FIGO, International Federation of Gynecology and Obstetrics; HR, hazard ratio; LVSI, lymphovascular space invasion; MSI, microsatellite instability; NSMP, no specific molecular profile; OR, odds ratio; OS, overall survival; p53abn, p53-abnormal; PFS, progression-free survival; POLEmut, POLE-mutated; RFS, recurrence-free survival.

**Table 1:** Distribution of molecular subtypes in included studies.

Author, Year	Ref.	*n*	POLEmut *n* (%)	MMRd *n* (%)	p53abn *n* (%)	NSMP *n* (%)
Betella, 2022	[1]	278	27 (9.7%)	77 (27.7%)	49 (17.6%)	125 (45.0%)
Arcieri, 2024	[2]	99	10 (10.1%)	30 (30.3%)	11 (11.1%)	48 (48.5%)
Han, 2024	[10]	161	19 (11.8%)	46 (28.6%)	28 (23.3%)	74 (46.0%)
Kobayashi-Kato, 2023	[14]	265	36 (13.6%)	70 (26.4%)	56 (21.1%)	103 (38.9%)
Talhouk, 2017	[16]	143	6 (4.2%)	30 (21.0%)	27 (18.9%)	80 (55.9%)
Kommoss, 2018	[17]	83	11 (13.3%)	22 (26.5%)	13 (15.7%)	37 (44.6%)
Stelloo, 2015	[18]	200	33 (16.5%)	38 (19.0%)	25 (12.5%)	104 (52.0%)
Berek, 2023	[19]	720	74 (10.6%)	161 (23.0%)	151 (21.6%)	314 (44.9%)
McCluggage, 2024	[20]	136	9 (6.6%)	25 (18.4%)	22 (16.2%)	80 (58.8%)
Vrede, 2022	[28]	393	33 (8.4%)	78 (19.8%)	72 (18.3%)	210 (53.4%)
Lee, 2025	[36]	345	0 (0%) *	81 (23.5%)	33 (9.6%)	231 (67.0%)
Cun, 2024	[37]	46	10 (21.7%)	18 (39.1%)	6 (13.0%)	12 (26.1%)
Luzarraga, 2024	[38]	658	47 (7.1%)	234 (35.5%)	95 (14.4%)	282 (42.8%)
TOTAL (all studies)		3527	315	910	588	1700
% (all studies)			8.9%	25.8%	16.7%	48.2%
TOTAL (excluding Lee, 2025)	[36]	3182	315	829	555	1469
% (excluding Lee, 2025)	[36]		9.9%	26.1%	17.4%	46.2%

NA: not available. * The study did not perform POLE sequencing. The weighted percentage for POLEmut was calculated excluding the study by Lee et al. (2025) [36], which did not perform POLE sequencing. When this cohort is excluded, the weighted frequency of POLEmut increases from 8.9% to 9.9% (315/3182 patients). The other subtype frequencies were recalculated accordingly: MMRd 26.1%, p53abn 17.4%, and NSMP 46.2%.

**Table 2:** Five-year survival rates by molecular subtype.

Author, Year	Ref.	Subtype	Overall Survival 5-Year (%)	Disease-Free/Progression-Free Survival 5-Year (%)
Han, 2024	[10]	POLEmut	NA	88.5%
		p53abn	NA	43.8%
Kobayashi-Kato, 2023	[14]	POLEmut	95.5%	92.7%
		p53abn	76.8% (hazard ratio)	74.9% (hazard ratio)
Zhao, 2025	[19]	POLEmut	99.2%	95.3%
		p53abn	NR	NR
Luzarraga, 2024	[38]	POLEmut	100%	97.8%
		MMRd	89.0%	81.9%
		p53abn	54.1%	33.1%
		NSMP	90.5%	78.8%
Vrede, 2022	[28]	POLEmut	95–100%	NA
		p53abn	41–90% (by grade)	NA
Kuroki, 2025	[39]	p53abn	70.7%	58.7%
		MMRd	84.3%	74.4%
		p53 wild-type	95.3%	83.4%

NA: not available; NR: not reported.

**Table 3:** Hazard ratios for survival by molecular subtype.

Author, Year	Ref.	Subtype	Hazard Ratio (95% CI)	Variable	Adjustment
Kobayashi-Kato, 2023	[14]	POLEmut	0.749 (0.55–0.98)	Progression-free survival	Multivariable
		p53abn	0.768 (0.60–0.94)	Overall survival	Multivariable
Luzarraga, 2024	[38]	p53abn	8.54 (2.0–36.3)	Peritoneal recurrence	Multivariable
		p53abn	6.51 (1.1–37.4)	Vaginal recurrence	Multivariable
		p53abn	3.13 (1.1–8.7)	Distant recurrence	Multivariable
		NSMP	2.35 (1.1–5.0)	Distant recurrence	Multivariable
Vrede, 2022	[28]	TP53 altered	1.76 (1.04–2.95)	Disease-specific survival	Multivariable
		POLE altered	0.16 (0.02–1.16)	Disease-specific survival	Multivariable
		MSI	0.51 (0.21–1.22)	Disease-specific survival	Multivariable
Kuroki, 2025	[39]	p53abn	0.71 (0.33–1.52)	Recurrence-free survival (VCB/C vs. RT)	Subgroup
Yin, 2025 (meta-analysis)	[40]	POLE	0.39 (0.25–0.60)	FIGO II-IV vs. I	Pooled
		CNH (p53abn)	3.05 (2.05–4.53)	FIGO II-IV vs. I	Pooled
		CNL (NSMP)	0.64 (0.54–0.75)	FIGO II-IV vs. I	Pooled
		MSI	1.04 (0.88–1.23)	FIGO II-IV vs. I	Pooled
		CNH	2.12 (1.72–2.62)	Lymphovascular invasion positive	Pooled
		CNH	3.06 (1.68–5.58)	Nodal metastasis	Pooled
		POLE	0.25 (0.08–0.79)	Nodal metastasis	Pooled
		CNL	0.31 (0.18–0.55)	Nodal metastasis	Pooled

CI: confidence interval. Hazard ratio <1 indicates better prognosis for POLEmut. Hazard ratio >1 indicates worse prognosis. VCB/C: vaginal brachytherapy + chemotherapy; RT: radiotherapy. CNH: copy-number high; CNL: copy-number low; MSI: microsatellite instability.

**Table 4:** Summary of key findings.

Finding	Result
Most frequent subtype	NSMP (48.2%)
Best prognosis	POLEmut (5-year overall survival >95%, hazard ratio <0.5)
Worst prognosis	p53abn (5-year disease-free survival 33–58%, hazard ratio >2.0)
Peritoneal recurrence risk factor	p53abn (odds ratio 8.54)
Vaginal recurrence risk factor	p53abn (odds ratio 6.51)
Distant recurrence risk factor	NSMP (hazard ratio 2.35) and p53abn (hazard ratio 3.13)
Risk reclassification	6–33% of patients
Molecular classification method	ProMisE (70.4% of studies)

## Data Availability

No new data were created or analyzed in this study. Data sharing is not applicable to this article.

## Supplementary Materials

The following supporting information can be downloaded at: https://www.mdpi.com/article/10.3390/cancers18203316/s1, Table S1: Quality assessment and risk of bias of all included studies (*n* = 50); Table S2: GRADE assessment of certainty of evidence; Table S3: PRISMA 2020 checklist.
