# Supplementary material for: Integrated Molecular Classification of Endometrial Cancer: Impact on Diagnosis, Prognosis, and Clinical Management—A Systematic Review
Source: Cancers (Basel). 2026 Oct 14;18(20):3316. doi: 10.3390/cancers18203316 (PMC13702350; doi:10.3390/cancers18203316)
Supplement: Supplementary file 1 [file cancers-18-03316-s001.zip › cancers-4583488-supplementary.pdf]

Table S1. Quality Assessment and Risk of Bias of All Included Studies (n = 50).

#	Study	Design	Tool	Score / Rating	Quality	Comments
1	Betella, 2022 [1]	Retrospective cohort	NOS	9/9	Good	—
2	Arcieri, 2024 [2]	Retrospective cohort	NOS	8/9	Good	No survival data; preprint
3	Siegel, 2023 [3]	Cancer statistics	N/A	—	—	Epidemiological data
4	Sung, 2021 [4]	GLOBOCAN statistics	N/A	—	—	Epidemiological data
5	Crosbie, 2022 [5]	Narrative review	Narrative appraisal	High	Good	Lancet seminar
6	Concin, 2021 [6]	Clinical practice guideline	AGREE II	High	Good	ESGO/ESTRO/ESP guideline
7	Bokhman, 1983 [7]	Historical study	N/A	—	—	Foundational classification
8	Gilks, 2013 [8]	Reproducibility study	N/A	—	—	Methodological study
9	Pecorelli, 2009 [9]	Clinical practice guideline (FIGO 2009)	N/A	—	—	Superseded by FIGO 2023
10	Han, 2024 [10]	Retrospective cohort	NOS	8/9	Good	No loss-to-follow-up reported
11	Guan, 2011 [11]	Reproducibility study	N/A	—	—	Methodological study

12	TCGA, 2013 [12]	Foundational genomic study	N/A	—	—	TCGA landmark study
13	McAlpine, 2021 [13]	IPD meta-analysis	AMSTAR-2	High	Good	Individual patient data; 294 patients
14	Kobayashi-Kato, 2023 [14]	Retrospective cohort	NOS	8/9	Good	Single-center; long time span
15	Talhouk, 2015 [15]	Retrospective cohort	NOS	8/9	Good	Discovery cohort; no external validation
16	Talhouk, 2017 [16]	Retrospective cohort	NOS	8/9	Good	Single-center; cases from 1983
17	Kommoss, 2018 [17]	Retrospective cohort	NOS	9/9	Good	Population-based; external validation
18	Stelloo, 2015 [18]	Retrospective cohort	NOS	8/9	Good	Multicenter; exploratory; limited follow-up
19	Berek, 2023 [19]	Clinical practice guideline	AGREE II	High	Good	FIGO 2023 staging
20	McCluggage, 2024 [20]	Position paper	Narrative appraisal	Moderate–High	Good	Critical appraisal of FIGO 2023
21	Espinosa, 2024 [21]	Critical review	Narrative appraisal	Moderate	Good	10 years of TCGA

22	Raffone, 2019 [31]	Meta-analysis	AMSTAR-2	Moderate	Good	Pooled data; >5,000 patients
23	León-Castillo, 2020 [29]	Ancillary analysis of RCT (PORTEC-3)	RoB 2	Some concerns	Good	Post-hoc; exploratory subgroups
24	León-Castillo, 2020 [30]	Characterization study	Narrative appraisal	Moderate	Good	Multiple-classifier EC
25	Oaknin, 2022 [50]	Clinical practice guideline	AGREE II	High	Good	ESMO guideline
26	Mirza, 2023 [32]	Randomized clinical trial (RUBY)	RoB 2	Low risk	Good	Phase III; primary endpoint met
27	Eskander, 2023 [33]	Randomized clinical trial (KEYNOTE-868)	RoB 2	Low risk	Good	Phase III; primary endpoint met
28	Orellana, 2023 [34]	Cost-effectiveness analysis	CHEERS	High	Good	Stage III EC; molecular testing
29	Page, 2021 [22]	PRISMA 2020 guideline	N/A	—	—	Reporting guideline
30	Wells, 2021 [23]	NOS tool	N/A	—	—	Quality assessment tool
31	Sterne, 2019 [24]	RoB 2 tool	N/A	—	—	Quality assessment tool

32	Shea, 2017 [25]	AMSTAR-2 tool	N/A	—	—	Quality assessment tool
33	Brouwers, 2010 [26]	AGREE II tool	N/A	—	—	Quality assessment tool
34	Husereau, 2013 [27]	CHEERS tool	N/A	—	—	Reporting guideline
35	Vrede, 2022 [28]	Retrospective multicenter cohort	NOS	9/9	Good	Multicenter; international; validation cohort
36	Uijterwaal, 2024 [45]	Retrospective cohort	NOS	7/9	Good	Stage IV; no independent prognostic value
37	Loverro, 2025 [48]	Retrospective cohort	NOS	7/9	Good	Stage IVB; single-center
38	Chen, 2025 [46]	Retrospective cohort (real-world)	NOS	7/9	Good	National database; testing rates
39	Bakshi, 2026 [41]	Retrospective cohort	NOS	6/9	Moderate	Single-center; no survival data
40	Timmermans, 2026 [49]	Retrospective cohort	NOS	7/9	Good	Danish cohort; NSMP high-grade
41	Zheng, 2025 [43]	Retrospective multicenter cohort	NOS	8/9	Good	Multicenter; nomogram; C-index 0.875
42	Zhu, 2025 [44]	Retrospective cohort	NOS	8/9	Good	Single-center; NEEC; complete follow-up

43	Tsolakidis, 2024 [42]	Retrospective cohort	NOS	7/9	Good	Single-center; 7.7% molecular testing
44	Davies, 2024 [47]	Prospective cohort	NOS	6/9	Moderate	Implementation study; no survival data
45	Gaffney, 2024 [35]	Narrative review	Narrative appraisal	Moderate	Good	FIGO 2023 evolution
46	Lee, 2025 [36]	Retrospective cohort	NOS	7/9	Good	Single-center; no POLE sequencing
47	Cun, 2024 [37]	Retrospective cohort	NOS	7/9	Good	Small sample; highly selected cohort
48	Luzarraga, 2024 [38]	Retrospective cohort	NOS	9/9	Good	Large sample; long follow-up; recurrence patterns
49	Yin, 2025 [40]	Meta-analysis	AMSTAR-2	Moderate	Good	Pooled data; >4,700 patients
50	Kuroki, 2025 [39]	Ancillary analysis of RCT (GOG-0249)	RoB 2	Some concerns		

Abbreviations: NOS, Newcastle-Ottawa Scale; RoB 2, Cochrane Risk of Bias tool for randomised trials (version 2); AMSTAR-2, A MeASurement Tool to Assess systematic Reviews (version 2); AGREE II, Appraisal of Guidelines for Research and Evaluation II; CHEERS, Consolidated Health Economic Evaluation Reporting Standards; N/A, not applicable.

Quality categories: NOS: Good (7–9 points), Fair (4–6 points), Poor (0–3 points). RoB 2: Low risk, Some concerns, High risk. AMSTAR-2: High, Moderate, Low, Critically low. AGREE II: High, Moderate, Low. CHEERS: High, Moderate, Low. Narrative appraisal: High, Moderate–High, Moderate, Low.

Note on non-assessable studies: Sixteen publications (references 3, 4, 7, 8, 9, 11, 12, 22, 23, 24, 25, 26, 27) were not formally assessed with quality assessment tools because they are not primary research studies or systematic reviews. These include epidemiological statistics, historical foundational studies, methodological reproducibility studies, superseded

guidelines, reporting guidelines, and quality assessment tools. Their inclusion in this systematic review is justified for context, methodology, and reference purposes, and they do not contribute primary outcome data to the synthesis.

Table S2. GRADE Assessment of Certainty of Evidence for Main Outcomes.

**Outcome**	**Number of studies**	**Study design**	**Risk of bias**	**Inconsistency**	**Indirectness**	**Imprecision**	**Publication bias**	**Certainty (GRADE)**
POLEmut associated with excellent prognosis (5-yr OS >95%)	8	Observational cohorts	Not serious	Not serious	Not serious	Not serious	Suspected	Moderate
p53abn associated with worst prognosis (5-yr DFS 33–58%)	8	Observational cohorts	Not serious	Not serious	Not serious	Not serious	Suspected	Moderate
p53abn as independent risk factor for peritoneal recurrence	1	Observational cohort	Serious	Not applicable	Not serious	Serious	Suspected	Low
p53abn as independent risk factor for vaginal recurrence	1	Observational cohort	Serious	Not applicable	Not serious	Serious	Suspected	Low
NSMP associated with distant recurrence	1	Observational cohort	Serious	Not applicable	Not serious	Serious	Suspected	Low
Molecular classification modifies risk group assignment	5	Observational cohorts	Not serious	Serious	Not serious	Not serious	Suspected	Low

(6–33%)								
FIGO 2023 improves prognostic discrimination vs FIGO 2009	3	Observational cohorts	Not serious	Serious	Not serious	Not serious	Suspected	Low
MMRd associated with intermediate prognosis (5-yr OS 80–90%)	8	Observational cohorts	Not serious	Not serious	Not serious	Serious	Suspected	Low
NSMP associated with intermediate prognosis (5-yr OS 78–90%)	8	Observational cohorts	Not serious	Not serious	Not serious	Serious	Suspected	Low

Abbreviations: DFS, disease-free survival; GRADE, Grading of Recommendations Assessment, Development and Evaluation; MMRd, mismatch repair deficiency; NSMP, no specific molecular profile; OS, overall survival; p53abn, p53 abnormal; POLEmut, POLE-mutated.

GRADE certainty levels: High, Moderate, Low, Very Low.

Assessment criteria: Observational cohort studies start as "Low" certainty. Certainty was upgraded to "Moderate" when consistent evidence from multiple studies supported a large, reproducible effect (POLEmut and p53abn prognostic associations). Certainty remained "Low" for outcomes based on single studies, heterogeneous results, or wide confidence intervals (recurrence patterns, FIGO 2023 comparison, MMRd/NSMP intermediate prognosis). Publication bias was suspected across all outcomes given the predominance of positive findings in the literature. No outcome was rated "High" due to the observational nature of the included studies and the lack of randomized evidence for the prognostic associations.

PRISMA

PRISMA 2020 Checklist

Table S3. PRISMA 2020 checklist.

Section and Topic	Item #	Checklist item	Location where item is reported
**TITLE**			
Title	1	Identify the report as a systematic review.	Title page (contains "A Systematic Review")
**ABSTRACT**			
Abstract	2	See the PRISMA 2020 for Abstracts checklist.	Abstract (structured: Objective, Methods, Results, Conclusion)
**INTRODUCTION**			
Rationale	3	Describe the rationale for the review in the context of existing knowledge.	Introduction – paragraphs 1-4
Objectives	4	Provide an explicit statement of the objective(s) or question(s) the review addresses.	Introduction – final paragraph
**METHODS**			
Eligibility criteria	5	Specify the inclusion and exclusion criteria for the review and how studies were grouped for the syntheses.	Methods – Eligibility Criteria
Information sources	6	Specify all databases, registers, websites, organisations, reference lists and other sources searched or consulted to identify studies. Specify the date when each source was last searched or consulted.	Methods – Search Strategy (PubMed, Web of Science, Scopus, Cochrane Library; searched December 2025, covering 2013–2025)
Search strategy	7	Present the full search strategies for all databases, registers and websites, including any filters and limits used.	Methods – Search Strategy (full PubMed strategy provided)
Selection process	8	Specify the methods used to decide whether a study met the inclusion criteria of the review, including how many reviewers screened each record and each report retrieved, whether they worked independently, and if applicable, details of automation tools used in the process.	Methods – Study Selection Process (two reviewers independently screened titles/abstracts

Section and Topic	Item #	Checklist item	Location where item is reported
			and full texts; disagreements resolved by consensus or third reviewer)
Data collection process	9	Specify the methods used to collect data from reports, including how many reviewers collected data from each report, whether they worked independently, any processes for obtaining or confirming data from study investigators, and if applicable, details of automation tools used in the process.	Methods – Data Extraction (two reviewers independently extracted data using a standardised Excel sheet; discrepancies resolved by consensus)
Data items	10a	List and define all outcomes for which data were sought. Specify whether all results that were compatible with each outcome domain in each study were sought (e.g. for all measures, time points, analyses), and if not, the methods used to decide which results to collect.	Methods – Data Extraction (survival outcomes: overall survival, disease-free survival, recurrence-free survival, disease-specific survival; hazard ratios; recurrence patterns)
	10b	List and define all other variables for which data were sought (e.g. participant and intervention characteristics, funding sources). Describe any assumptions made about any missing or unclear information.	Methods – Data Extraction (study characteristics, population features, molecular classification method, stage migration, therapeutic implications, cost-effectiveness, barriers)
Study risk of bias assessment	11	Specify the methods used to assess risk of bias in the included studies, including details of the tool(s) used, how many reviewers assessed each study and whether they worked independently, and if applicable, details of automation tools used in the process.	Methods – Quality

PRISMA

PRISMA 2020 Checklist

Section and Topic	Item #	Checklist item	Location where item is reported
			Assessment and Risk of Bias (Newcastle-Ottawa Scale for cohorts, RoB 2 for trials, AMSTAR-2 for reviews, AGREE II for guidelines, CHEERS for cost-effectiveness; two reviewers independently; consensus)
Effect measures	12	Specify for each outcome the effect measure(s) (e.g. risk ratio, mean difference) used in the synthesis or presentation of results.	Methods – Data Extraction; Results – Tables 2–3 (hazard ratios, odds ratios, 5-year survival rates)
Synthesis methods	13a	Describe the processes used to decide which studies were eligible for each synthesis (e.g. tabulating the study intervention characteristics and comparing against the planned groups for each synthesis (item #5)).	Methods – Data Synthesis; Results (studies grouped by theme: subtype distribution, survival, recurrence, stage migration, therapy)
	13b	Describe any methods required to prepare the data for presentation or synthesis, such as handling of missing summary statistics, or data conversions.	Methods – Data Synthesis (narrative synthesis; no de novo meta-analysis performed)
	13c	Describe any methods used to tabulate or visually display results of individual studies and syntheses.	Methods – Data Synthesis; Results – Tables 1–4 and Figures 1–2
	13d	Describe any methods used to synthesize results and provide a rationale for the choice(s). If meta-analysis was performed, describe the	Methods – Data Synthesis (no de

Section and Topic	Item #	Checklist item	Location where item is reported
		model(s), method(s) to identify the presence and extent of statistical heterogeneity, and software package(s) used.	novo meta-analysis; pooled estimates from Yin et al., 2025)
	13e	Describe any methods used to explore possible causes of heterogeneity among study results (e.g. subgroup analysis, meta-regression).	Not performed; discussed in limitations
	13f	Describe any sensitivity analyses conducted to assess robustness of the synthesized results.	Not performed
Reporting bias assessment	14	Describe any methods used to assess risk of bias due to missing results in a synthesis (arising from reporting biases).	Not formally assessed; acknowledged in Discussion – Strengths and Weaknesses
Certainty assessment	15	Describe any methods used to assess certainty (or confidence) in the body of evidence for an outcome.	GRADE assessment performed; Table S2.
**RESULTS**			
Study selection	16a	Describe the results of the search and selection process, from the number of records identified in the search to the number of studies included in the review, ideally using a flow diagram.	Results – first paragraph and Figure 1 (PRISMA flow diagram).
	16b	Cite studies that might appear to meet the inclusion criteria, but which were excluded, and explain why they were excluded.	Figure 1 (reasons for exclusion).
Study characteristics	17	Cite each included study and present its characteristics.	Results – text and Tables 1–4
Risk of bias in studies	18	Present assessments of risk of bias for each included study.	Table S1 (Supplementary) – Quality Assessment.
Results of individual studies	19	For all outcomes, present, for each study: (a) summary statistics for each group (where appropriate) and (b) an effect estimate and its precision (e.g. confidence/credible interval), ideally using structured tables or plots.	Tables 2–3; Figure 2 (Forest Plot).
Results of syntheses	20a	For each synthesis, briefly summarise the characteristics and risk of bias among contributing studies.	Results – text
	20b	Present results of all statistical syntheses conducted. If meta-analysis was done, present for each the summary estimate and its precision (e.g. confidence/credible interval) and measures of statistical heterogeneity. If comparing groups, describe the direction of the effect.	Table 3 (pooled estimates from Yin et al.)
	20c	Present results of all investigations of possible causes of heterogeneity among study results.	Not performed
	20d	Present results of all sensitivity analyses conducted to assess the robustness of the synthesized results.	Not performed

PRISMA

PRISMA 2020 Checklist

Section and Topic	Item #	Checklist item	Location where item is reported
Reporting biases	21	Present assessments of risk of bias due to missing results (arising from reporting biases) for each synthesis assessed.	Not assessed (acknowledged as potential limitation in Discussion)
Certainty of evidence	22	Present assessments of certainty (or confidence) in the body of evidence for each outcome assessed.	Table S2 (GRADE assessment)
**DISCUSSION**			
Discussion	23a	Provide a general interpretation of the results in the context of other evidence.	Discussion – Summary of Main Results and Results in the Context of Published Literature
	23b	Discuss any limitations of the evidence included in the review.	Discussion – Strengths and Weaknesses
	23c	Discuss any limitations of the review processes used.	Discussion – Strengths and Weaknesses
	23d	Discuss implications of the results for practice, policy, and future research.	Discussion – Implications for Practice and Future Research
**OTHER INFORMATION**			
Registration and protocol	24a	Provide registration information for the review, including register name and registration number, or state that the review was not registered.	Methods – Study Design (states: "The review protocol was not registered in PROSPERO.")
	24b	Indicate where the review protocol can be accessed, or state that a protocol was not prepared.	Methods – Study design
	24c	Describe and explain any amendments to information provided at registration or in the protocol.	Not applicable
Support	25	Describe sources of financial or non-financial support for the review, and the role of the funders or sponsors in the review.	Funding Statement (no funding received)
Competing interests	26	Declare any competing interests of review authors.	Conflict of Interest Statement (none

Section and Topic	Item #	Checklist item	Location where item is reported
			declared)
Availability of data, code and other materials	27	Report which of the following are publicly available and where they can be found: template data collection forms; data extracted from included studies; data used for all analyses; analytic code; any other materials used in the review.	Methods – Data Synthesis (data available upon reasonable request)

From: Page MJ, McKenzie JE, Bossuyt PM, Boutron I, Hoffmann TC, Mulrow CD, et al. The PRISMA 2020 statement: an updated guideline for reporting systematic reviews. BMJ 2021;372:n71. doi: 10.1136/bmj.n71. This work is licensed under CC BY 4.0. To view a copy of this license, visit https://creativecommons.org/licenses/by/4.0/
